# Propagule Limitation, Disparate Habitat Quality, and Variation in Phenotypic Selection at a Local Species Range Boundary

**DOI:** 10.1371/journal.pone.0089404

**Published:** 2014-04-09

**Authors:** Kara A. Moore, Maureen L. Stanton

**Affiliations:** Department of Evolution and Ecology, Center for Population Biology, University of California Davis, Davis, California, United States of America; Stanford University, United States of America

## Abstract

Adaptation to novel conditions beyond current range boundaries requires the presence of suitable sites within dispersal range, but may be impeded when emigrants encounter poor habitat and sharply different selection pressures. We investigated fine-scale spatial heterogeneity in ecological dynamics and selection at a local population boundary of the annual plant *Gilia tricolor*. In two years, we planted *G. tricolor* seeds in core habitat, margin habitat at the edge of the local range, and exterior habitat in order to measure spatial and temporal variation in habitat quality, opportunity for selection, and selection on phenotypic traits. We found a striking decline in average habitat quality with distance from the population core, yet some migrant seeds were successful in suitable, unoccupied microsites at and beyond the range boundary. Total and direct selection on four out of five measured phenotypic traits varied across habitat zones, as well as between years. Moreover, the margin habitat often exerted unique selection pressures that were not intermediate between core and exterior habitats. This study reveals that a combination of ecological and evolutionary forces, including propagule limitation, variation in habitat quality and spatial heterogeneity in phenotypic selection may reduce opportunities for adaptive range expansion, even across a very local population boundary.

## Introduction

A central focus of ecology is to understand how the distributions of species take shape and change over time. Empirical studies conducted at scales ranging from meters (e.g. [1]–[2]) to hundreds of kilometers (e.g. [3]–[4]) across the boundaries of species' distributions have identified two major limiting ecological factors: (1) the intersection of the realized niche with local environmental heterogeneity and (2) restricted spatial dispersal of successful, well-adapted migrants. In the last three decades, a spate of theoretical models has built upon this ecological foundation to ask how divergent selection and gene flow may limit niche expansion and shape distributional boundaries (reviewed in [5]–[6]). Even so, simultaneous empirical analyses of the ecological and evolutionary conditions that can facilitate range expansion or enforce range conservatism remain relatively rare.

At the geographic scale, transplant studies have found that some distributional limits are set by barriers to dispersal [7]–[9], while others are significantly defined by climate [10]–[11], biotic interactions [12]–[13], and/or environmental quality [3],[14]. Within species ranges, experiments in which individuals are transplanted just meters beyond a species' local population boundaries have often shown that local population limits are set by transitions between contrasting micro-environmental regimes [2],[15]–[20], rather than by restricted dispersal.

Spatial population dynamics interact with population genetics in shaping both geographic and local range limits over generations. Local adaptation to lower-quality habitats within the range of seed and pollen dispersal from established populations is well documented (e.g. [21]–[22]). For example, adaptation to novel conditions is a notable feature of many plant invasion fronts (e.g. [23]–[26]). Alternatively, distributional boundaries may be conserved where migrants lack genetic variation suited to novel selection pressures [5], [27]–[29]. Experiments focused specifically on disentangling the ecological and evolutionary drivers that together constrain range margins of natural populations are relatively rare, but a few studies conducted at larger geographic scales have shown that experimental migrants moved to or across their species range limit encounter very different selection pressures from those in central portions of the range [30]–[32].

The spatial scale and the magnitude of habitat heterogeneity are critically important to evaluating the suite of factors that contribute to range limits. According to the “maladapted migrant” hypothesis, steeper gradients in population density and reproductive rates (and, by inference, habitat quality) should lead to more asymmetric core-to-edge dispersal, while more dramatic differences in selection will generate higher adaptive hurdles for emigrants from core habitats (e.g. [33]–[35]). From an empirical standpoint, adaptation to novel conditions at or beyond population margins should be less likely if: (1) suitable sites are relatively uncommon within the dispersal range of core populations, (2) non-core subpopulations occupy lower-quality sites that are likely to be demographic “sinks”, and/or (3) selection regimes differ dramatically between core and non-core habitats. To our knowledge, no one has empirically tested for all of these conditions in any single system.

We used a field experiment on the annual plant, *Gilia tricolor*, in two years to investigate ecological and evolutionary factors expected to impede or facilitate expansion of a local range boundary. Whereas previously Baack et al. [36], determined how ecological factors limit the local distribution of *Gilia* within the mixed serpentine and non-serpentine landscape, our aim was to address key components of the maladapted migrant hypothesis by characterizing potential limitations in propagule supply, the distribution of habitat quality, and variation in selection regimes across the local boundary of this natural plant population. We asked:

1. Are sites at or beyond the population boundary propagule-limited?
2. How does the quality of habitat encountered by seeds vary among core, margin, and exterior habitats?
3. Across the population boundary, is there spatial or temporal variation in the opportunity for selection on the variation in phenotype and relative fitness?
4. Does phenotypic selection vary spatially and/or temporally across the population boundary?
5. Is there a general pattern in the strength of phenotypic selection over multiple traits such that it is stronger in one habitat zone than another?

By focusing on a local population boundary in two years, our aim was to characterize variation in habitat quality and natural selection at spatial and temporal scales relevant to seed dormancy, seed dispersal and pollen dispersal. We hypothesized that selective differences between the habitat zones might be driven by higher competition in more productive non-serpentine microsites exterior to the population core, resulting in selection for early emergence to preempt emergence of exotic grasses, and for greater height to gain access to more light within the dense grassland canopy. In contrast, we hypothesized that selection in the more serpentine-influenced population core would favor smaller plants capable of completing reproduction and senescing prior to early soil dry-down. We envisioned two possible outcomes for selection regimes at the population margin– as either distinct or intermediate between those in core and exterior habitats.

## Materials and Methods

### Study system

This research was conducted with the permission of the University of California Donald and Sylvia McLaughlin Natural Reserve in Napa and Lake Counties, California, USA. Field studies did not involve any threatened or endangered species

**Figure 1:**
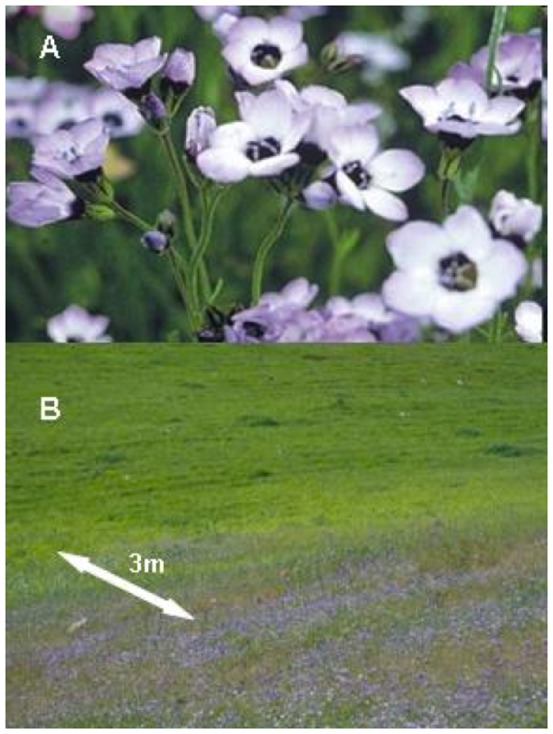
Gilia tricolor at the study site. (**a**) Close-up photograph of a group of closely growing *Gilia tricolor* individuals in core habitat. (**b**) Photograph of the down-slope edge of the study population, showing the rapid transition in *G. tricolor* density across the local population boundary from the population core to exterior habitat zone.

*Gilia tricolor* (Benth., Polemoniaceae, hereafter “*Gilia*”) is an annual plant native to California, USA, and common throughout much of the California Floristic Provence. Within its range it often occurs in conspicuous, dense patches bounded by sharp declines in population density (Fig. 1). At our study site, *Gilia* occurs in a dense patch (∼0.25 ha) within an annual-dominated, mixed serpentine and non-serpentine grassland plant community. This patch and its sharp population boundary provide an excellent opportunity to address the ecological and evolutionary processes in operation at local range limits. Our study population occurs on a southwest–facing, variably steep slope (15–40°), well within the north central distribution of the species (latitude 38.825519 longitude −122.347211, elevation 450 m). This study population and the habitat zones at its boundary (described below) are characteristic of other *Gilia* population boundaries in grasslands both locally and regionally. We conducted our experimental study in 2008 and 2010, two growing seasons that differed substantially in the timing and amount of precipitation (Fig. S1). There was nearly 30% more precipitation in 2010 (2008: 20.04 mm; 2010: 28.02 mm), and rains continued through May. In contrast, in 2008 there was negligible precipitation after early February (Fig. S1).

Despite marked year-to-year fluctuations in aboveground *Gilia* density, the location of the *Gilia* population boundary has remained remarkably stable across the past 12 years. This might in part be driven by the presence of a dormant seed bank of unknown age; buried seeds have been observed to emerge at very low rates after 4 or more years (Moore and Stanton, unpublished data). Within our study site we demarcated 3 habitat zones based on the spatial and temporal consistency of adult plant density: (1) “core” habitat supports dense, contiguous *Gilia* with few unoccupied sites >0.1 m^2^; (2) “margin” habitat is characterized by spatially and/or temporally variable *Gilia* densities, with few unoccupied sites >0.25 m^2^; and (3) “exterior” habitat has very few *Gilia*, if any, even at scales >1.0 m^2^, yet is plausibly within the dispersal range of both seed and pollen. The transition between habitat zones is steep, with exterior and core habitats often separated by <3 m (Fig. 1). The core zone is influenced by weak to moderate serpentine soil chemistry, whereas the exterior zone has soil that is mostly non-serpentine in composition. We initially identified potential margin habitat in Fall 2007 based on a 2001 demarcation of population “periphery” and “edge” zones across the population boundary by Baack et al. [36]. In each subsequent season we resurveyed density in quadrats placed throughout the study site. Based on fluctuations in *Gilia* density between 2007 and 2012, 7 quadrat locations (out of 60 total) were reassigned from either the “core” or “exterior” category to the “margin” because their density varied substantially between years. In the margin, and to a lesser degree in the core, density of *Gilia* in any single year was spatially patchy at the 1-m^2^ scale. Rare *Gilia* individuals have been observed in exterior habitat well beyond the margin in each growing season.

To assess the underlying correlates of *Gilia* habitat zones, soil samples were collected at five locations in each habitat zone in 2003 for the Baack et al. [36] study. Six additional samples were collected in exterior zone locations in 2011. Samples were collected at the soil surface to a depth of approximately 5 cm, dried and sent for laboratory nutrient analysis. The 2003 samples were analyzed at the University of California Davis DANR Analytical Laboratory. The 2011 samples were analyzed at A & L Western Agricultural Laboratories in Modesto, California. There were no distinguishable differences between years for samples collected in the exterior zone.

### Experimental seed migration

We conducted a planting experiment over the 2007–2008 and 2009–2010 growing seasons to determine the fate of experimental migrant seeds and to compare phenotypic selection on these migrants in core, margin, and exterior habitats. We refer to these as the 2008 and 2010 planting experiments, since *Gilia* flowers in the spring following fall or winter emergence. In September 2007, we established 60 sets of paired 0.0625 m^2^ experimental plots. Plots in each pair were 10 cm apart and were randomly located along and between 6 transects perpendicular to the population boundary, ranging downslope from core *Gilia* habitat into the productive, invaded grassland below in which *Gilia* is largely absent. Transects were randomly spaced along a baseline running through the middle of the core habitat zone. Average distance between transects was approximately 10 m. We placed 16 plot pairs in core habitat, 14 pairs in margin habitat and 30 pairs in exterior habitat. Plots were distributed along transects and to their east and west via selection of random coordinates. A minimum of 2 m separated each plot in each direction. For the 2008 planting experiment, *Gilia* seeds were sown into one plot, while a paired plot was used to observe *Gilia* that naturally emerged from the seed bank. We replanted a subset of 25 of the 2008 experiment seeding plot locations in November 2009 for the 2010 planting experiment with 0.8982 m^2^ experimental plots. Exterior plots in which *Gilia* had not emerged from the seed bank or sown seeds in 2008 or 2009 were not used again; all core and margin and the majority of exterior plots were replanted in 2010. Plots were located such that there were 5 in core habitat, 11 in margin habitat and 14 in exterior habitat. In both years, margin habitat plots ranged 0.9–7.5 m and exterior habitat plots ranged 4.0–31.1 m from core habitat.

We affixed seeds to wooden toothpicks with water-soluble glue prior to field planting, making it possible to distinguish experimental seedlings from natural emergents [2], [36]–[38]. In each year we sowed surplus seeds available from greenhouse hand-crosses that were conducted for other experiments. Typically, few seeds were available from any given cross. Seeds for the 2008 study were mostly from crosses between core- and margin-origin lineages. Seeds used in the 2010 experiment came from crosses involving all three habitat zones, although there were few exterior-origin parents due to the dearth of exterior plants. Prior to sowing, all seeds were stored at room temperature in dry conditions in our laboratory. In 2008, 2 seeds were attached to each toothpick, and 10 toothpicks were sown per plot by placing them in pre-drilled planting holes ∼4 mm^2^. This method creates nearly no disturbance around the planted seeds. In 2010, 3 seeds were attached to each toothpick and 150 toothpicks were sown per plot. By varying plot size, toothpick density was kept nearly the same between years (2008: 160/m^2^, 2010: 167/m^2^), well below the density of naturally occurring *Gilia* in the majority of core habitat. Seeds were positioned just below ground level in plots that were otherwise undisturbed. For the 2008 season we planted 1,194 seeds on 598 toothpicks in 60 small plots; for the 2010 season we planted 12,834 seeds on 4,278 toothpicks in 30 large plots.

We calculated the proportion emergence from seeds sown into each plot. Where more than a single plant emerged at the same toothpick, the emergent closest to the toothpick was kept and others were clipped at the soil level. In >95% of cases in which more than one seedling emerged at a toothpick, emergents appeared on the same census day. Subsequent studies on this species in which 4–5 seeds glued onto single toothpicks have emerged throughout a field experiment have reduced our concerns that gluing multiple seeds per toothpick might limit emergence rates or seedling success.

On each emergent plant we measured lifetime fruit production, phenological traits, and size traits. Beginning shortly after planting and continuing through the growing season, we measured: days from sowing to emergence, longest leaf length at flowering, days from sowing to first bud and first flower, days from emergence to senescence, and lifetime fruit production. We selected these traits because of their likely importance to plant success, observed variation across the study site, and the feasibility of measuring them on the majority of short-lived individuals, despite asynchronous emergence and growth. For annual plants with a very short growing season, such as *Gilia*, the timing of emergence and flowering relative to the end of winter precipitation and the beginning of summer heat are critical to performance. Traits associated with larger overall size may indicate higher growth rate or a longer effective growing season, both of which may be influenced by local environmental conditions. We harvested the vast majority of individuals (>98% in each year) when they started to senesce. A few plants were harvested prior to senescence, but by this time field conditions had deteriorated markedly and the vast majority of *Gilia* plants had already senesced and been harvested; there would be no additional fruit production beyond this point. Harvested plants were dried at 40°C for at least 48 hours and weighed. The longest internode, a size metric of plant stem elongation and competitive response, was measured on dried plants. We estimated post-emergence fitness as the total number of fruits produced. There was very little variation in days to first flower within habitat zones, and so this variable was not used in selection analyses. In each survey year the number of naturally occurring *Gilia* plants in each plot was tallied at early flowering.

### Analyses

Analyses and data visualization were conducted in R (version 2.13.2) using the packages car, glmmADMB, and lmer.

#### Variation in habitat quality and availability

We used MANOVA to test for changes in a suite of inter-related soil mineral properties and ANOVA to test for changes in soil water and annual aboveground productivity across the habitat boundary. We used ANOVA to test for variation among habitat zones in the density of naturally occurring *Gilia* and the percent emergence of experimental seed for each year. We used a generalized linear mixed model with a zero-inflated Poisson distribution to test for differences in post-emergence fitness of individuals emerging from planted seeds across the population boundary; plot was included as a random effect. Where appropriate, we used Tukey's HSD test to compare means between each pair of habitat zones. For naturally occurring *Gilia* density, the tally of plants in each plot that emerged from the seed bank, the two study years were not statistically compared because of slight differences in the timing of our density counts.

To test for propagule limitation, we compared *Gilia* density in the 60 seeded and non-seeded plots in 2008 to compare the effects of seed supplementation across habitat zones. We calculated density in each plot as the sum of naturally occurring *Gilia* individuals and those produced from experimental migrant seeds, and used separate ANOVAs to test for effects of seed addition on density within each habitat zone. Statistical significance was assessed after Bonferroni correction for multiple tests (*N* = 3).

#### Variation in the opportunity for selection

Variance in phenotypic traits provides the raw material for natural selection, and the variance in relative fitness among individuals (also known as the “opportunity for selection”; [39]) reflects the maximum potential strength of total phenotypic selection. Adaptation at or beyond a population's margins could be constrained by low levels of variance in key phenotypes and low relative fitness variation. To determine whether variance in fitness and phenotype differed across habitat zones and years, we conducted Levene's tests (1) on relative fitness (with habitat zone, year and their interaction as effects), and (2) on each of five phenotypic traits (emergence day, midseason leaf length, senescence day, final internode length, and final biomass) in which habitat zone was the sole effect for each year. Significant differences in variance represent changes in the opportunity, or raw material, for selection across habitat zones and/or years. We assessed correlations between all traits with Pearson product-moment tests.

#### Variation in the magnitude and direction of selection

We compared the strength and direction of phenotypic selection on each trait between years and across habitat zones, in models either with or without final biomass as a covariate. We first used a series of general mixed models to estimate the relationship between a single trait and relative post-emergence fitness [40]. In these models, conducted within each habitat zone and year, the regression coefficient (*S*) for the standardized trait value was interpreted as estimating the overall magnitude and direction of selection on that trait, estimated total selection (*S_TS_*). For each trait-specific selection analysis, post-emergence fitness was relativized to its mean within habitat zone and year. Traits were standardized to have a mean of zero and a standard deviation of 1.0 within habitat zone and year (e.g. [40]). Because data on phenotypic traits were collected throughout the lives of individual plants, sample sizes were often much smaller for traits measured after significant mortality had occurred (see Table S4 and S5 for sample sizes). Data for each trait were available for a unique subset of the total emergents, and so relative fitness was calculated separately for each combination of trait, year, and habitat zone. For these reasons, it was not possible to jointly evaluate directional selection on all measured traits.

Next, to explore whether the observed relationship between a given focal trait and relative post-emergence fitness could simply reflect variation in environmental quality, we evaluated an additional mixed model for each trait (again, within each habitat zone and year) in which plant final biomass, a strong indicator of microsite quality, was included as a covariate [41]–[42]. We calculated a second estimate of selection (*S_-b_*) on each trait as the regression coefficient for that trait when plant size was included in the model. Because biomass is a strong determinant of fitness in this system, shows considerable correlation with other measured size traits and is associated with both the abiotic and biotic environmental gradients, its inclusion as a covariate provides a meaningful estimate of direct selection on the focal trait. We stress that this metric controls just for selection on biomass and its correlated traits, and thus only approximates actual direct selection on the focal trait. Our confidence in these estimates of direct selection is strongly supported by a recent review in which Kingsolver et al. [43] found that indirect selection due to correlated traits is generally very low.

To assess the response of relative post-emergence fitness to each standardized phenotypic trait, habitat zone, year and their interactions, we first assessed general trends in the spatial and temporal variation in both total and estimated direct selection via a generalized linear model to assess interactions between standardized trait value, habitat zone and year. A significant three-way interaction, for example, would indicate that selection was more variable among habitat zones in one year than in the other. Because of low sample sizes, especially in exterior plots in 2008, final plant traits (longest internode and biomass) were excluded from some analyses. For analysis of each trait, we first used maximum likelihood to fit general linear mixed models with all fixed effects and their interactions. Plot was included as a random factor in each model. We used chi-square likelihood ratio (*LR*) tests to determine the significance of each fixed effect by testing the effects of single-term deletions from the full model [44].

We then conducted two sets of bootstrapped selection models to generate robust estimates of the magnitude and direction of total and estimated direct selection. First, for each trait, we bootstrapped models testing the interaction between year and standardized trait. Second, for each trait, we bootstrapped models testing the interaction between habitat zone and standardized trait. In each case, we bootstrapped the focal model 10,000 times and included plot as a random effect. We considered directional phenotypic selection to be statistically significant when the 95% bootstrapped confidence interval on the selection differential did not overlap zero. We conservatively assessed the significance of variation in selection either between years or among habitat zones within each year based on non-overlapping 95% confidence intervals. We applied this same bootstrapping procedure to models estimating either total or direct selection for each trait.

#### Overall strength of phenotypic selection

We tested for differences in overall selection by collectively assessing variation in the mean values for selection on each trait within year and habitat zone. We used means of the significant bootstrapped estimates of total selection (those with 95% confidence intervals that did not overlap zero) within each year, for each habitat zone, to evaluate spatial variation in the overall strength of total phenotypic selection across all five traits. First, within each habitat zone and year, we calculated the absolute value of the selection coefficient (|*S_TS_*|) from the bootstrapped means of each of the five-univariate trait models to estimate the overall strength of total selection on each trait. Second, we used MANOVA to test for differences in the overall strength of total selection, |*S_TS_*|, across habitat zones and years, as estimated by the |*S_TS_*| values from each trait-specific full selection model. Tukey's HSD tests on were used to test *a posteriori* for differences between habitat zones within each year.

## Results

### Variation in habitat characteristics

Gradual but significant changes in edaphic and vegetation attributes occur perpendicular to the sharp *Gilia* population boundary, as the ultramafic, serpentinite hillside soils characterizing core habitat grade irregularly into valley-bottom soils below. The MANOVA based on eight soil properties revealed significant variation among habitat zones (Organic matter, P, pH, K, Ca, NO3-N, Zn, Mn; no pairs correlated *r*>0.66; Table S1). Moving from core to exterior habitats, the decreasing influence of serpentine soil was suggested by changes in soil texture and appearance, as well as a decline in proximity to ultramafic rock outcrops, but there was no difference in soil Ca:Mg between core and margin soil samples. We also found no difference in % soil water among the three habitat zones during peak *Gilia* emergence in 2008 (Tables S2 and S3). Annual productivity of the plant community, as measured by weight or depth of accumulated thatch, increased dramatically from core to exterior (Tables S2 and S3). Productivity was approximately two times greater in exterior than in core habitat in each year; this contrast was statistically significant in 2010 (Tables S2 and S3).

### Variation in habitat quality

**Figure 2:**
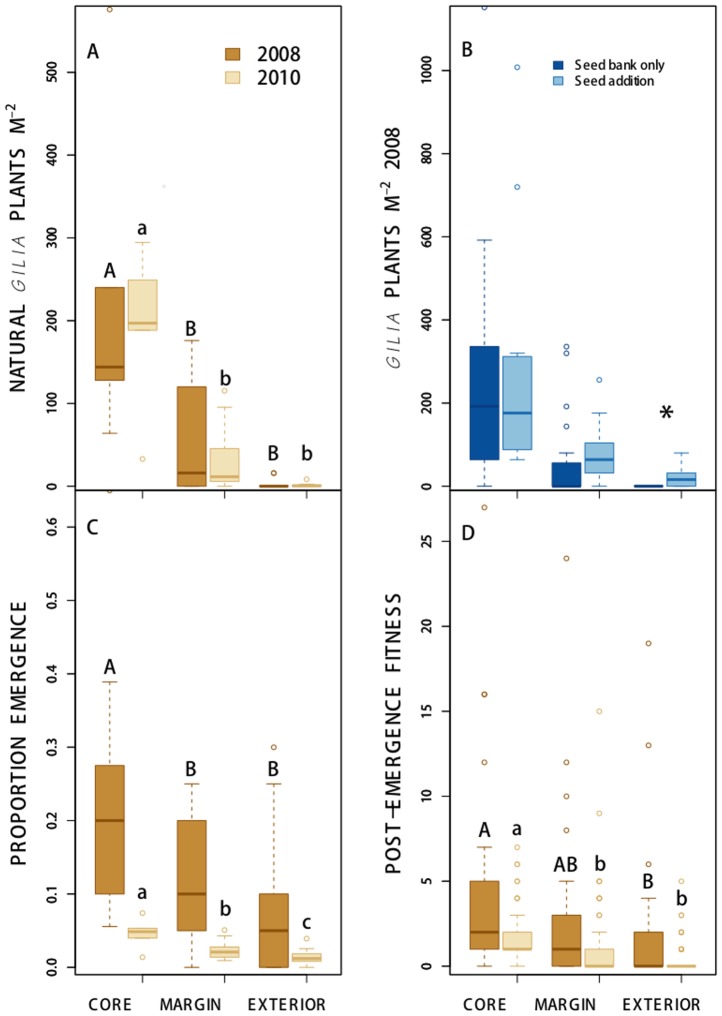
Spatial and temporal variation in Gilia tricolor. Difference in *Gilia triocolor* (**a**) natural density, (**b**) density response to seeding treatment, and (**c**) emergence and (**d**) post-emergence fitness of seeded plants in three habitat zones that span a local population boundary. Differences in the naturally occurring density of *G. tricolor* plants/m^2^ across habitat zones in 30 plots were compared in 2008 and in 2010. To evaluate propagule limitation, densities of *G. tricolor* in 60 paired seeded and non-seeded plots were compared in each habitat zone in 2008; * indicates the significant difference in *G. tricolor* density due to sowing treatment in the exterior habitat only. In plots (a), (c), and (d), differences between 2008 and 2010 for each response in 30 seeded plots among habitat zones are shown. Post-emergence fitness is defined as the number of fruits of *G. tricolor* experimental plants in three habitat zones in each season. Within each year, habitat zones not sharing a letter differed in *a posteriori* Tukey's test comparisons, *P*<0.05; upper case letters refer to contrasts within 2008, lower case letters refer to contrasts within 2010.

As expected, core habitat had significantly greater natural *Gilia* density than either margin or exterior habitat in each year (Fig. 2a). Margin habitat had spatially variable, intermediate density, and a few rare individuals occurred in exterior plots (0.13 plants/m^2^, Fig. 2a). In 2008, natural density in the core was 33% lower than in 2010.

The 2008 seeding experiment demonstrated clear propagule limitation beyond the population boundary, despite the close proximity of core and exterior habitats (Fig. 2b). Addition of experimental migrant seeds significantly increased density in exterior sites (*F*_1,58_ = 29.921, *P* = 0.0010), to 1.6 plants/m^2^. Sowing additional seeds had no significant effect on *Gilia* densities in core and margin habitats.

Habitat quality experienced by planted seeds consistently declined from core to margin and then exterior habitats (Fig. 2c–d). However, the decrease in habitat quality beyond the core, measured as both pre- and post-emergence fitness of experimental seeds, was not as precipitous as the natural density gradient. Some migrant seeds succeeded in suitable microsites beyond the core habitat in each year (Fig. 2). Migrant seeds to exterior plots successfully produced fruits as far as 31.1 m from core habitat in 2008 and as far as 26.4 m away in 2010.

Both habitat zone and year had significant overall effects on proportion emergence and post-emergence fitness (Emergence: habitat type *F*_2,84_ = 9.7936, *P* = 0.0001; year *F*_1,84_ = 25.8778, *P*<0.001; Fitness: habitat zone deviance = 16.969, *P* = 0.0022, year: deviance = 10.084, *P* = 0.0179; interactions not significant). Despite greater precipitation and a longer wet season in 2010 (Fig. S1), proportion emergence and post-emergence fitness were significantly greater in 2008 (Fig. 2c, d). In 2008, emergence was significantly greater in the core than in the other two habitat zones; there was marginally greater emergence in the margin habitat zone than in the exterior (*F*_1,47_ = 1.68, *P* = 0.0966, Fig. 2c). As we expected, post-emergence fitness was significantly greater in the core than in exterior habitat in both years, but in each year, some seeded migrants were able to reproduce in the exterior zone (Fig. 2d). The emergence and post-emergence fitness of planted seeds in 2010 was low in all habitat zones (Fig. 2c–d). In 2010, when conditions for *Gilia* were very poor, there was significant variation in emergence rate among all habitat zones (Fig. 2c), yet some seeds emerged and reproduced in both margin and exterior zones (Fig. 2d).

### Variation in phenotype and relative fitness

The general phenotypic response of experimental plants to the three habitat zones was for individuals to emerge later, but be larger and taller in core habitat, and to emerge earlier and decrease in size along the gradient from margin to exterior habitat (Tables S4 and S5). Internode length was significantly greater in core habitat than in exterior habitat in both years, whereas leaf length did not vary among habitat zones in either year (Tables S4 and S5). Phenology traits showed little correlation with plant biomass (Tables S6–S7). Leaf length was inconsistently correlated with plant biomass, whereas internode length was positively correlated with plant biomass in all habitat zones in each year (Tables S6–S7). Within habitat zones in each year there were only two other correlations between traits with *r*>0.65– internode length and leaf length were positively correlated in the core and margin zones in 2008 (both *r*<0.80).

To make meaningful comparisons of phenotypic selection on focal traits across the population boundary, we first had to demonstrate that there was opportunity for selection, estimated as substantial variance in relative fitness, in all habitat zones. Indeed, we found that in both years, there was similar variance in relative post-emergence fitness of experimental plants across the three habitat zones. Levene's tests for variance differences in relative fitness between habitat zones within each year were not significant (2008: *F*_2,121_ = 0.1570, *P* = 0.8549; 2010: *F*_2,277_ = 1.3636, *P* = 0.2574). There were also comparable levels of phenotypic variance across habitat zones within years (*P*>0.0500 in Levene's tests on each trait within each year), whereas trait variances differed significantly between years (*P≤*0.0500 for all traits). Spatially consistent variance in relative fitness and phenotype suggests that there was both ample opportunity and raw material for selection on experimental plants in all habitat zones spanning the population boundary.

### Temporal and spatial variation in phenotypic selection

Across years and habitat zones, phenotypic selection on *G. tricolor* plants is extremely dynamic. For leaf length, internode length and biomass, we detected consistent spatial variation in total directional selection across the two years (Table 1). Although estimates of total and direct selection on phenotypic traits ranged substantially across the natural *Gilia* population boundary, patterns of selection tended to vary more in magnitude than in direction among core, margin and exterior sites (Table 2). Only for biomass did we document statistically significant year-to-year variation in the relative strength of total selection among habitat zones. Total phenotypic selection on each of the five measured traits varied significantly between years (Table 1, Table 2, and Table S8). Estimated direct selection, accounting for variation in individual biomass, also differed between years for some traits (Table 3 and Table 4).

**Table 1:** Results of five separate generalized linear mixed models analyzing variation in estimated total phenotypic selection (*S_TS_*) on five traits of *Gilia tricolor* experimental plants across three habitat zones and two years.

	Trait*habitat zone*year		Trait*year		Trait*habitat zone	
Response	*LR*	*P*	*LR*	*P*	*LR*	*P*
Emergence day	0.45	0.7988	2.25	**0.0403**	4.21	0.3248
Senescence day	5.91	0.0521*	30.12	**<0.0001** *	5.80	0.0550
Leaf length	2.54	0.2807	5.85	**0.0156**	36.72	**<0.0001** *
Longest internode	3.21	0.0732* §	6.24	**0.0125** * §	11.41	**0.0007** * §
Biomass	10.58	**0.0011** §	10.58	**0.0011** §	56.96	**<0.0001** §

Separate series of models were conducted for each of five plant traits analyzing interactions between trait, habitat zone, and year. Significance of fixed effects was assessed by likelihood ratio tests (*LR*) using single-term deletions; plot was a random effect in each model. Fitness was relativized and traits were standardized within habitat zone and year. Effects significant at the *P*<0.05 level are shown in bold.

§ denotes where the exterior habitat zone was excluded from analyses because of limited sample size in 2008.

*denotes tests that showed significant variation in direct phenotypic selection (*β_DS_*) when biomass was included as a covariate.

**Table 2:** The mean strength and direction of estimated total phenotypic selection (*S_TS_*) on five traits of *Gilia tricolor* within year and habitat zone.

	2008						2010					
	Core		Margin		Exterior		Core		Margin		Exterior	
**Response**												
Emergence Day	−0.26	a	−0.22	a	−0.51	a	**0.20**	a	0.09	a	−0.18	a
Senescence Day	**0.77**	a	**0.72**	a	0.16	a	**0.42**	a	**1.08**	b	**1.25**	ab
Leaf Length	**0.84**	a	**1.11**	a	**1.14**	a	**0.27**	a	**1.10**	b	**0.86**	b
Longest Internode	0.01	a	−0.39	a	0.21	a	**0.51**	a	**0.76**	a	**1.02**	a
Biomass	**1.00**	a	**1.40**	a	**0.30**	b	**0.42**	a	**1.08**	a	**1.25**	b

Selection coefficients were calculated by separate bootstrapped separate generalized linear mixed models predicting relative fitness from standardized values of each trait. Plot was a random effect in each model. Selection coefficients in bold have 95% confidence intervals that do not overlap zero; habitat zones with the same letter are within overlapping confidence intervals (see supporting information).

**Table 3:** The mean strength and direction of estimated direct phenotypic selection (*S_-b_*), selection on the focal trait while biomass was included as a covariate, on four traits of *Gilia tricolor* within year and habitat zone.

	2008				2010					
	Core		Margin		Core		Margin		Exterior	
**Response**										
Emergence Day	**1.52**	a	0.00	b	0.22	a	**0.61**	b	**0.61**	ab
Senescence Day	**1.08**	a	0.16	b	**0.40**	a	**1.47**	b	0.39	a
Leaf Length	**1.06**	a	0.06	b	**0.20**	a	**0.52**	a	−0.08	a
Longest Internode	**1.53**	a	−0.28	b	−0.18	a	**0.56**	a	0.22	a

Selection coefficients were calculated by separate bootstrapped separate generalized linear mixed models predicting relative fitness from standardized values of each trait. Plot was a random effect in each model; standardized biomass was included as a covariate. Selection coefficients in bold have 95% confidence intervals that do not overlap zero; habitat zones with the same letter are within overlapping confidence intervals (see supporting information). Due to limited sample size, *S_-b_* was not estimated for the exterior habitat zone in 2008.

**Table 4:** The mean strength and direction of estimated total phenotypic selection (*S_TS_*) and direct selection (*S_-b_*) in two experimental years on each of five phenological traits of *Gilia tricolor*.

	*S_TS_*				*S_-b_*			
	2008		2010		2008		2010	
**Response**								
Emergence Day	−0.29	a	0.33	a	−0.02	a	**0.41**	a
Senescence Day	−0.12	a	**0.90**	b	0.04	a	**0.48**	b
Leaf Length	**1.04**	a	−0.43	b	**0.41**	a	−0.43	a
Longest Internode	**0.83**	a	−0.27	b	**0.23**	a	−0.19	a
Biomass	**1.05**	a	−0.27	b	—	—	—	—

Mean selection coefficients were calculated by separate bootstrapped generalized linear mixed models that predict relative fitness from standardized values of each trait. Plot was a random effect in each model. For *S_-b_, standardized biomass was included as a covariate*. Selection coefficients in bold have 95% confidence intervals that do not overlap zero; years with the same letter are within overlapping confidence intervals (see supporting information).

*Gilia* migrants from densely populated core sites to margin and exterior sites will often encounter variable, and in some cases, novel selection environments. However, we did not document the strong changes in the direction of selection on measured traits that would contribute to adaptive tradeoffs across the population boundary. The selection landscape showed subtle shifts between 2008 and 2010. When we controlled for final biomass, estimated direct selection, *S*_-b_, favored late emergence in the core (but not the margin) in 2008. In contrast, *S*_-b_ for late emergence was weakest in the core in 2010 (Table 3, Table S9). Late-senescing plants achieved minimal gains in relative fitness in exterior sites in 2008, but were favored in all habitat zones in 2010, and also experienced the strongest fitness advantage in exterior and margin sites in that year (Table 2). Estimated direct selection *S*_-b_, on emergence day showed a similar pattern of variation among habitat zones (Table 3, Table S9), indicating that selection for delayed senescence was not being driven simply by the accumulation of more biomass. As expected, most size traits (biomass, leaf length and internode length) were associated with greater lifetime fitness in this annual plant; larger plants achieved greater lifetime fitness in all zones and years. However, averaged across all traits, the magnitude of *S*_TS_ ranged from 0.3 (in the exterior) to 1.4 (in the margin) in 2008, and from 0.42 (in the core) to 1.25 (in the exterior) in 2010. Comparing total selection (*S*_TS_) to estimated direct selection (*S*_-b_) among habitat zones is particularly revealing for internode length and leaf length, two traits that are usually highly correlated with biomass (Tables S6 and S7). In 2010, plants with long internodes achieved significantly greater lifetime fitness in all habitat zones (Table 2), but accounting for biomass variation revealed more spatial variability in estimated *S*_-b_ on internode length (Table 3, Table S9). Similarly, whereas *S*_TS_ for long leaves was consistently positive across habitat zones in 2008, inclusion of biomass in the model revealed that leaf length conferred a direct fitness advantage in just the core in 2008 and in the core and margin in 2010.

Directional selection in margin sites was often not intermediate between that estimated in core and exterior habitat zones, indicating that margin habitat may be a unique selection environment, rather than a transitional stepping-stone for population expansion. Of 10 possible comparisons for *S*_TS_ (five traits in 2 years), margin habitat exerted intermediate directional selection in only 50% of the cases (Table 2). In 2010 (the only year for which sample sizes are sufficient to estimate *S*_-b_ in the exterior habitat zone), the *S*_-b_ gradient in margin habitat was beyond the core-to-exterior range for all four traits (Table 3).

Selection on phenotypic traits differed markedly between the 2008 and 2010 seasons. Across all habitat zones, there was significant total and estimated direct selection for greater plant size and length traits in 2008, but selection on these traits was not significant in 2010 and tended to be negative (Table 4). In 2010, there was both total and estimated direct selection for later senescence (Table 4), suggesting that in this year later senescence conferred an advantage beyond its contribution to greater overall plant size. Similarly, later emergence was advantageous in 2010, indicating that plants benefited from starting and completing their life cycle later in that dry season (Table 4).

### Overall strength of selection on measured traits

**Figure 3:**
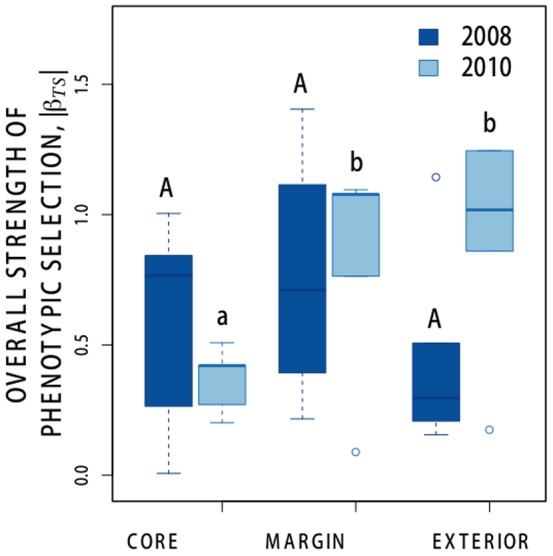
The overall strength of estimated total phenotypic selection, as measured by mean |*S_TS_*| from univariate models of selection on five traits of *Gilia tricolor* experimental plants in three habitat zones spanning a local population boundary in the 2008 (dark bars) and 2010 (light bars) seasons. Within each year, habitat zones not sharing a letter differed in *a posteriori* Tukey's test comparisons, *P*<0.05; upper case letters refer to contrasts within 2008, lower case letters refer to contrasts within 2010.

Summed across the five measured traits, the overall strength of total phenotypic selection (|*S_TS_*|, from selection models on individual traits) varied significantly across the habitat zone gradient (*F_2_* = 5.862, *P* = 0.0116; Fig. 3), but there was no significant difference in the overall strength of estimated *S_TS_* between years (*F_1_* = 0.0651, *P* = 0.3991). This effect was driven by significantly stronger overall total phenotypic selection in 2010 in both the margin and exterior zones, compared with core habitat (Fig. 3). The overall strength of total phenotypic selection did not vary significantly among habitat zones in 2008.

## Discussion

### Overview

Our results clearly demonstrate that dispersal limitation reduces the ability of the *Gilia tricolor* study population to expand into habitable sites just meters beyond its margin, which, while sharp and consistent, does not simply demarcate an abrupt transition from favorable to unassailable habitat. Rather, despite a striking decline in average environmental quality moving from the core to exterior habitat zone, our experiment identified rare suitable sites that were unoccupied beyond the population margin. Adding seeds to exterior habitat increased *Gilia* density, albeit spottily, in exterior sites, indicating that propagule limitation limits the exposure, and potentially adaptation, of this population to suitable microsites beyond the current boundary.

In addition to this fine-scale propagule limitation, we found spatiotemporal patterns in habitat quality and selection that could further reduce the potential for local adaptive expansion into the exterior zone. There were also substantial differences in weather between years, and temporal differences in other factors such as species interactions might also contribute to year-to-year variation in *Gilia* population dynamics. Even so, natural *Gilia* density and success, as well as the emergence and post-emergence fitness of experimental *Gilia* transplants, were consistently greatest in core habitat and generally declined with distance from the core (Fig. 2). Although the raw material for adaptation, estimated as levels of phenotypic variance and the opportunity for selection (variance in relative fitness) did not vary substantially across the population boundary, we observed significant spatial variation in phenotypic selection on the traits of plants emerging from experimental migrant seeds (Fig. 3, Tables 2 and 3). These results suggest that in addition to the formidable constraint of propagule limitation and small populations of emergent plants, migrant seeds would experience generally poor conditions and distinct selection regimes at the population margin and in the exterior zone.

### Restricted propagule availability beyond the population core

Even at the very fine spatial scales over which our experiment was conducted, we found that inadequate natural migration to the exterior zone in two years, in concert with low fitness in most exterior microsites, significantly limits *Gilia* local distribution. This finding suggests that propagule limitation may be a persistent ecological limitation to expansion of *Gilia* in favorable microsites beyond its current local range boundary. Consistent presence of extremely sparse *Gilia* individuals in exterior habitat is likely the long-term result of propagule flow down-slope across the population boundary from dense, highly fecund individuals in core habitat (Fig. 2a–b). However, natural dispersal into rare, suitable exterior zone microsites is apparently insufficient to compensate for lower average emergence rates, low post-emergence fitness, and strong selective pressures on emerging seeds in exterior sites beyond the current population boundary. In contrast, there was no propagule limitation in margin sites at the population boundary; seed addition did not significantly increase *Gilia* density within margin microsites, even though we seeded at a density more than twice that found naturally in these locations.

### Barriers to adaptation?

Our findings suggest that adaptive range expansion at this *Gilia* population boundary faces considerable challenges. Consistently high seed and pollen production in core habitat, in concert with strong declines in both density and average fecundity in margin and exterior sites, produce conditions in which asymmetric gene flow across the *Gilia* population boundary is likely. In this case, gene flow could easily swamp local selection. Non-core habitats were on average much lower in quality for *Gilia* than sites in the population core. Moreover, selection regimes differed between core and non-core habitats in 2008 and 2010. These factors conform to theoretical studies that have found niche conservatism to be fostered by asymmetric gene flow from common and/or high-quality core habitats into sparsely populated marginal habitats characterized by distinct selection pressures (reviewed in [6]).

Despite these constraints, we cannot rule out that this *G. tricolor* population could adapt to margin and exterior habitat over time, even given the observed spatial and temporal differences in selection size and phenological traits. Although there were sharp declines in emergence and post-emergence fitness across the population boundary, both natural and experimental migrants were occasionally able to establish and reproduce in non-core locations in both years. Although experimental migrants were largely maladapted to exterior microsites, a few migrants were able to succeed in these sites, despite the fact that they differed greatly in productivity and soil characteristics in comparison to core or margin habitat. On the whole, *Gilia* exterior habitat appears to be a demographic sink that is unable to maintain positive growth in isolation, and in which phenotypic selection is often stronger than in core habitat (Fig. 3). Still, if immigration is consistently sustained at a low rate by dispersal from core and margin, there may be opportunity for locally favorable variants that succeed in favorable exterior microsites to increase gradually in frequency over time [45]. Furthermore, although there were significant spatial differences in selection, we rarely observed a significant change in the direction of total or estimated direct selection on any trait across the population boundary (Tables 2 and 3). Finally, it is possible that seed dormancy and specific germination cueing could change the distribution of habitat qualities and selection pressures experienced by migrants, for example by allowing them to avoid unfavorable microhabitats or seasons [46]–[47].

### Unique conditions at the local range boundary

Current theoretical models for adaptation at range limits generally focus on core and exterior habitats, and as such do not consider the possibility that margin habitats may be qualitatively distinct from either core or exterior habitats. Our results show that the margin habitat at this *Gilia* population boundary has unique properties, and is not simply an intermediate “stepping stone” between core and exterior habitat. Experimental migrants to margin habitats met a selective environment that differed significantly from core habitat in the strength of estimated direct selection on all four traits in 2008, and in both phenological traits in 2010 (Tables 3). In 2010, the only year in which sample sizes allowed us to estimate direct phenotypic selection in all three-habitat types, phenotypic selection gradients in margin sites fell outside the range of estimated selection gradients for core and exterior sites for all traits we measured. When the strength of selection was pooled within year, we found that margin and exterior habitats tended to exert stronger overall selection on migrants in some years (Fig. 3).

### Does spatiotemporal variation in selection and habitat quality foster niche conservatism?

Spatial or temporal variation in selection pressures, such as that found at our *Gilia* population boundary, may limit adaptive expansion when a given trait is highly advantageous in some microsites or years, and not so in others [48]. Three factors suggest that spatiotemporal habitat quality variation could contribute to the conservation of this local *Gilia* population boundary. First, we found both strong significant variation in the *Gilia* density between core and margin habitat zones and significant temporal variation in *Gilia* density in core habitat between years (Figs. 1 and 2). We suspect that temporal variation in the density of core habitat quality is driven by the low water-holding capacity of serpentine soils. In wet years, core habitat on serpentine soil may offer a respite from competition, yet may remain sufficiently moist for emergence and post-emergence success. In dry years, serpentine soils may be doubly stressful [36], [49]. Second, given the temporal and spatial variation in microsite suitability across this population boundary, appropriate germination cueing that allows seeds to predict microsite quality should be under very strong selection, and could fundamentally alter the balance between niche conservation and expansion. In this study, we did not find a pattern of strong selection on emergence timing. However, constrained by logistic feasibility, our approach was not sufficiently temporally fine-grained to be able to detect adaptive matching to sporadic, rainy intervals. If *Gilia* indeed lacks adaptive emergence cueing, pre-emergence and early seedling mortality could be strong limiting factors at the population boundary, and could alter the effective temporal variability experienced by plants in core, margin, and exterior habitats. Additional information on seed dormancy, germination cueing, and the spatial distribution of suitable microsites will be important in future studies of plant adaptation at range boundaries. Third, the competitive gradient across the *Gilia* population boundary might compound selective differences between core and exterior habitat. In models, range boundaries can form along shallower environmental gradients where migrants encounter greater interspecific competition [50]–[51]. All of these factors could act with propagule limitation to inhibit adaptation at plant population boundaries, and merit further study.

### Conclusions

We found propagule limitation, reduced habitat quality and spatial and temporal shifts in selection pressures experienced by migrant seeds together reduce the opportunity for range expansion at this local population boundary. Migration beyond the local range boundary appears to be limited in more than a single season, and seeds that do migrate face challenging habitat conditions and experience spatial and temporal differences in selection patterns on multiple traits. Seed addition demonstrated that limited propagule supply can contribute to even very local range boundaries when suitable microsites are rare. Although this boundary type, grading from serpentine into non-serpentine grassland, is regionally common, it is likely that at other population boundaries (e.g. the oak woodland boundary discussed in [36]), the relative importance of dispersal, competition, and phenotypic selection on different traits may vary. At our study site, we identified specific spatial differences in phenotypic selection between core, margin and exterior habitat zones that might impede adaptation to conditions at and beyond the population core. In one year, the overall strength of selection was significantly greater at and beyond the population margin, suggesting that adaptation to conditions beyond the population core may be more challenging in some years than in others.

To our knowledge, this is one of the few studies to measure spatial and temporal variation in habitat quality and phenotypic selection using experimental plants distributed across a range boundary. Additional research that includes genetically structured plantings of experimental migrants is ongoing, and will allow testing for the existence of genetically based fitness trade-offs across the *Gilia* population boundary.

## Supporting Information

Figure S1 **Biweekly seedling emergence of experimental** ***Gilia tricolor*** **plants (bars) and biweekly precipitation (dashed line) for overwinter growing seasons ending in 2008 and 2010.** Seedling surveys were conducted every 7–14 days, weather permitting.
(TIFF)

Table S1 **Variation in soil organic and mineral properties across three habitat zones spanning a local population boundary of** ***Gilia tricolor.***
(DOCX)

Table S2 **Variation in the main effect of habitat zone on three environmental characteristics at experimental plots located in three habitat zones spanning a local population boundary of** ***Gilia tricolor*** **.**
(DOCX)

Table S3 **Variation in sample size, mean, and standard deviation of plot characteristics across three habitat zones panning a local population boundary of** ***Gilia tricolor*** **.**
(DOCX)

Table S4 **Variation in five phenotypic traits of experimental** ***Gilia tricolor*** **plants across three habitat zones spanning a local population boundary in 2008.**
(DOCX)

Table S5 **Variation in five phenotypic traits of experimental** ***Gilia tricolor*** **plants across three habitat zones spanning a local population boundary in 2010.**
(DOCX)

Table S6 **Pearson product-moment correlations between biomass and four phenotypic traits measured on experimental** ***Gilia tricolor*** **plants across three habitat zones spanning a local population boundary in 2008.**
(DOCX)

Table S7 **Pearson product-moment correlations between biomass and four phenotypic traits measured on experimental** ***Gilia tricolor*** **plants across three habitat zones spanning a local population boundary in 2010.**
(DOCX)

Table S8 **Bootstrapped confidence intervals for the strength and direction of estimated total phenotypic selection (** ***S_TS_*** **) on five traits of** ***Gilia tricolor.***
(DOCX)

Table S9 **Bootstrapped confidence intervals for the strength and direction of estimated direct phenotypic selection (** ***S_-b_*** **) on five traits of** ***Gilia tricolor*** **.**
(DOCX)
