# Supplementary figures and images for: Propagule Limitation, Disparate Habitat Quality, and Variation in Phenotypic Selection at a Local Species Range Boundary
Source: PLoS One. 2014 Apr 9;9(4):e89404. doi: 10.1371/journal.pone.0089404 (PMC3981700; doi:10.1371/journal.pone.0089404)

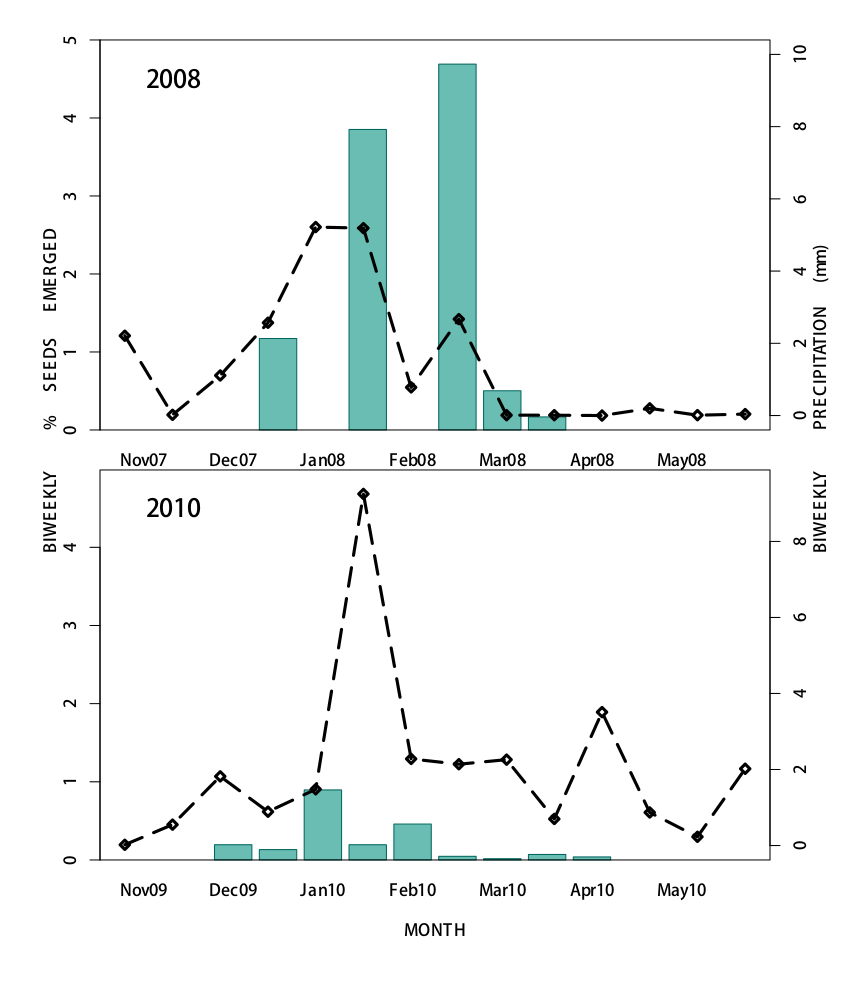

Supplement: Figure S1 — Biweekly seedling emergence of experimental Gilia tricolor plants (bars) and biweekly precipitation (dashed line) for overwinter growing seasons ending in 2008 and 2010. Seedling surveys were conducted every 7–14 days, weather permitting. (TIFF) [file pone.0089404.s001.tif]
