# Supplementary material for: Propagule Limitation, Disparate Habitat Quality, and Variation in Phenotypic Selection at a Local Species Range Boundary
Source: PLoS One. 2014 Apr 9;9(4):e89404. doi: 10.1371/journal.pone.0089404 (PMC3981700; doi:10.1371/journal.pone.0089404)
Supplement: Table S1 — Variation in soil organic and mineral properties across three habitat zones spanning a local population boundary of Gilia tricolor. (DOCX) [file pone.0089404.s002.docx]

**Table S1.** Variation in soil organic and mineral properties across three habitat zones spanning a local population boundary of *Gilia tricolor.*

|  |  | **Core** (*N*=5) | | **Margin** (*N*=5) | | **Exterior** (*N*=11) | |
| --- | --- | --- | --- | --- | --- | --- | --- |
|  |  | 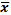 | ***se*** | 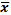 | **se** | 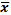 | ***se*** |
| * | % organic matter | 3.54 | 0.25 | 3.58 | 0.38 | 3.48 | 0.19 |
| * | P (Weak Bray, ppm) | 2.96 | 1.01 | 6.84 | 1.26 | 8.44 | 1.17 |
| * | pH | 7.00 | 0.08 | 7.02 | 0.05 | 7.06 | 0.06 |
| * | K (ppm) | 213.26 | 24.25 | 256.72 | 26.23 | 241.44 | 19.09 |
|  | Mg (ppm) | 2147.60 | 137.21 | 2055.40 | 317.36 | 2055.19 | 223.56 |
| * | Ca (ppm) | 1139.28 | 383.81 | 1352.30 | 440.14 | 1459.18 | 337.26 |
|  | CEC (meq/100g) | 24.16 | 1.20 | 24.46 | 1.15 | 25.05 | 0.88 |
| * | NO_3_-N (ppm) | 6.34 | 0.11 | 6.10 | 0.10 | 3.73 | 0.86 |
|  | SO_4_-S (ppm) | 8.00 | 3.67 | 11.80 | 2.89 | 8.00 | 1.91 |
| * | Zn (ppm) | 0.48 | 0.07 | 1.60 | 1.08 | 0.74 | 0.30 |
| * | Mn (ppm) | 13.40 | 1.91 | 11.76 | 1.05 | 7.62 | 1.32 |
|  | Fe (ppm) | 24.78 | 3.21 | 25.16 | 1.98 | 17.62 | 1.64 |
|  | Cu (ppm) | 0.78 | 0.04 | 1.26 | 0.16 | 0.82 | 0.06 |
|  | B (ppm) | 0.30 | 0.04 | 0.46 | 0.05 | 0.34 | 0.05 |

Core habitat is consistently occupied. Margin habitat shows greater spatial and/or temporal heterogeneity in occupancy and exterior habitat is occupied rarely. Soil attributes included in a MANOVA to test for differences between habitat zones are marked with a *. These were selected to be the most biologically meaningful set of soil attributes not correlated greater than *r* = 0.65.
