# Supplementary material for: Propagule Limitation, Disparate Habitat Quality, and Variation in Phenotypic Selection at a Local Species Range Boundary
Source: PLoS One. 2014 Apr 9;9(4):e89404. doi: 10.1371/journal.pone.0089404 (PMC3981700; doi:10.1371/journal.pone.0089404)
Supplement: Table S2 — Variation in the main effect of habitat zone on three environmental characteristics at experimental plots located in three habitat zones spanning a local population boundary of Gilia tricolor . (DOCX) [file pone.0089404.s003.docx]

**Table S2.** Variation in the main effect of habitat zone on three environmental characteristics at experimental plots located in three habitat zones spanning a local population boundary of *Gilia tricolor*.

|  |  | **% Soil water** | | | | **Thatch (g)** | | | | **Thatch depth (cm)** | | | |  |
| --- | --- | --- | --- | --- | --- | --- | --- | --- | --- | --- | --- | --- | --- | --- |
|  |  | **2008** | |  | | **2008** | |  | | **2010** | |  | |  |
|  | ***df*** | | ***F*** | | ***P*** | | ***F*** | | ***P*** | | ***F*** | | ***P*** | |
| Habitat zone | 2, 27 | 0.0344 | | 0.9663 | | 5.3939 | | **0.0107** | | 18.697 | | **<0.0001** | |  |

Core habitat is consistently occupied. Margin habitat shows greater spatial and/or temporal heterogeneity in occupancy and exterior habitat is occupied rarely. Effects significant to the *P* = 0.0500 level or less are shown in bold. Percent soil water was measured gravimetrically adjacent to each plot in March of 2008. Also in 2008, an aboveground vegetation sample was collected in a 0.0625-m^2^ area adjacent to each plot as an estimate of site productivity early and late in the growing season. This sample was dried at 40° C for 48 hours then weighed. In April of 2010, thatch depth was measured at 6 random sites within each plot.
