# Supplementary material for: Propagule Limitation, Disparate Habitat Quality, and Variation in Phenotypic Selection at a Local Species Range Boundary
Source: PLoS One. 2014 Apr 9;9(4):e89404. doi: 10.1371/journal.pone.0089404 (PMC3981700; doi:10.1371/journal.pone.0089404)
Supplement: Table S3 — Variation in sample size, mean, and standard deviation of plot characteristics across three habitat zones panning a local population boundary of Gilia tricolor . (DOCX) [file pone.0089404.s004.docx]

**Table S3.** Variation in sample size, mean, and standard deviation of plot characteristics across three habitat zones panning a local population boundary of *Gilia tricolor*.

|  |  | **% Soil water** | | | **Thatch (g)** | | | **Thatch depth (cm)** | | |
| --- | --- | --- | --- | --- | --- | --- | --- | --- | --- | --- |
|  | \|  \|  \|  \|  \| \| --- \| --- \| --- \| --- \|  \|  \| \| --- \| | **2008** | | | **2008** | | | **2010** | | |
|  | ***N*** | 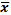 |  | ***se*** | 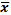 |  | ***se*** | 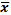 |  | ***se*** |
| Core | 5 | 6.2 | a | 1.7 | 13.4 | a | 2.7 | 1.2 | a | 0.3 |
| Margin | 9 | 5.8 | a | 0.6 | 19.3 | a* | 5.4 | 1.6 | a | 0.1 |
| Exterior | 16 | 6.0 | a | 0.4 | 30.9 | b* | 2.6 | 2.3 | b | 0.1 |

Within each year, habitat types not sharing a letter differed in *a posteriori* Tukey’s test comparisons, *P* < 0.05. * marks a difference of marginal significance, *P*=0.0651. See Table S1 for additional detail on environmental metrics.
