# Supplementary material for: Propagule Limitation, Disparate Habitat Quality, and Variation in Phenotypic Selection at a Local Species Range Boundary
Source: PLoS One. 2014 Apr 9;9(4):e89404. doi: 10.1371/journal.pone.0089404 (PMC3981700; doi:10.1371/journal.pone.0089404)
Supplement: Table S4 — Variation in five phenotypic traits of experimental Gilia tricolor plants across three habitat zones spanning a local population boundary in 2008. (DOCX) [file pone.0089404.s005.docx]

**Table S4.** Variation in five phenotypic traits of experimental *Gilia tricolor* plants across three habitat zones spanning a local population boundary in 2008.

|  | **Emergence Day** | | | | **Leaf Length** | | | | **Senescence Day** | | | | | **Longest Internode** | | | | | **Biomass** | | | | | | | |  |  |
| --- | --- | --- | --- | --- | --- | --- | --- | --- | --- | --- | --- | --- | --- | --- | --- | --- | --- | --- | --- | --- | --- | --- | --- | --- | --- | --- | --- | --- |
|  | ***N*** | 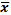 |  | ***σ^2^*** | ***N*** | 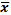 |  | ***σ^2^*** | | ***N*** | 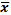 |  | ***σ^2^*** | | ***N*** | 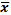 |  | ***σ^2^*** | | ***N*** | | 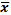 | |  | ***σ^2^*** | | |  |
| Core | 38 | 67.1 | a | 469.7 | 30 | 1.3 | a | 0.5 | | 38 | 127.9 | a | 606.4 | | 26 | 21.6 | a | 91.5 | | 26 | | 18.3 | | a | 419.6 | | |  |
| Margin | 42 | 64.7 | a | 473.0 | 32 | 1.1 | a | 0.6 | | 42 | 131.1 | a | 474.1 | | 29 | 16.4 | ab | 75.1 | | 29 | 10.5 | | b | | | 335.8 | | |
| Exterior | 44 | 62.6 | a | 302.4 | 23 | 1.3 | a | 0.9 | | 44 | 132.3 | a | 276.2 | | 7 | 13.2 | b | 22.3 | | 7 | | 6.7 | | b | 19.7 | | |  |

Plants occupying habitat zones sharing the same letter were not significantly different with respect to the focal trait in 2008 (*P* > 0.05). See Table 1 for significance of main effects.
