# Supplementary material for: Propagule Limitation, Disparate Habitat Quality, and Variation in Phenotypic Selection at a Local Species Range Boundary
Source: PLoS One. 2014 Apr 9;9(4):e89404. doi: 10.1371/journal.pone.0089404 (PMC3981700; doi:10.1371/journal.pone.0089404)
Supplement: Table S5 — Variation in five phenotypic traits of experimental Gilia tricolor plants across three habitat zones spanning a local population boundary in 2010. (DOCX) [file pone.0089404.s006.docx]

**Table S5.** Variation in five phenotypic traits of experimental *Gilia tricolor* plants across three habitat zones spanning a local population boundary in 2010.

|  | | **Emergence Day** | | | | | **Leaf Length** | | | | | **Senescence Day** | | | | | | **Longest Internode** | | | | | **Biomass** | | | |
| --- | --- | --- | --- | --- | --- | --- | --- | --- | --- | --- | --- | --- | --- | --- | --- | --- | --- | --- | --- | --- | --- | --- | --- | --- | --- | --- |
|  | | ***N*** | | 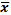 |  | ***σ^2^*** | ***N*** | | 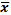 |  | ***σ^2^*** | ***N*** | | 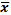 |  | ***σ^2^*** | ***N*** | 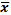 | |  | ***σ^2^*** | ***N*** | 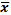 | |  | ***σ^2^*** |
| Core | | 96 | | 62.0 | a | 671.6 | 76 | | 0.6 | a | 0.2 | 95 | | 82.0 | a | 1297.4 | 44 | 16.9 | | a | 52.6 | 44 | 3.7 | | a | 20.9 |
| Margin | | 104 | | 55.0 | ab | 588.0 | 68 | | 0.6 | a | 0.2 | 104 | | 119.0 | ab | 1539.0 | 39 | 14.3 | | ab | 55.6 | 42 | 4.2 | | a | 73.1 |
| Exterior | | 80 | | 52.0 | b | 386.7 | 38 | | 0.5 | a | 0.1 | 80 | | 109.8 | b | 1274.7 | 20 | 9.9 | | c | 39.2 | 22 | 1.0 | | a | 3.5 |

Plants occupying habitat zones sharing the same letter were not significantly different with respect to the focal trait in 2010 (*P* > 0.05). See Table 1 for significance of main effects.
