# Supplementary material for: Propagule Limitation, Disparate Habitat Quality, and Variation in Phenotypic Selection at a Local Species Range Boundary
Source: PLoS One. 2014 Apr 9;9(4):e89404. doi: 10.1371/journal.pone.0089404 (PMC3981700; doi:10.1371/journal.pone.0089404)
Supplement: Table S6 — Pearson product-moment correlations between biomass and four phenotypic traits measured on experimental Gilia tricolor plants across three habitat zones spanning a local population boundary in 2008. (DOCX) [file pone.0089404.s007.docx]

**Table S6.** Pearson product-moment correlations between biomass and four phenotypic traits measured on experimental *Gilia tricolor* plants across three habitat zones spanning a local population boundary in 2008.

|  | **Emergence Day** | | | **Leaf Length** | | | **Senescence Day** | | | **Longest Internode** | | |
| --- | --- | --- | --- | --- | --- | --- | --- | --- | --- | --- | --- | --- |
|  | ***N*** | ***r*** | ***P*** | ***N*** | ***r*** | ***P*** | ***N*** | ***r*** | ***P*** | ***N*** | ***r*** | ***P*** |
| Core | 26 | -0.2057 | 0.3134 | 25 | 0.7209 | **<0.0001** | 26 | -0.0594 | 0.7732 | 26 | 0.7661 | **<0.0001** |
| Margin | 29 | -0.2539 | 0.1839 | 29 | 0.8668 | **<0.0001** | 29 | -0.5105 | **0.0047** | 29 | 0.7709 | **<0.0001** |
| Exterior | 7 | -0.6270 | 0.1318 | 6 | -0.3277 | 0.5261 | 7 | 0.1306 | 0.7802 | 7 | 0.9503 | **0.0010** |

Correlations significant at *P* < 0.05 are shown in bold.
