# Supplementary material for: Propagule Limitation, Disparate Habitat Quality, and Variation in Phenotypic Selection at a Local Species Range Boundary
Source: PLoS One. 2014 Apr 9;9(4):e89404. doi: 10.1371/journal.pone.0089404 (PMC3981700; doi:10.1371/journal.pone.0089404)
Supplement: Table S7 — Pearson product-moment correlations between biomass and four phenotypic traits measured on experimental Gilia tricolor plants across three habitat zones spanning a local population boundary in 2010. (DOCX) [file pone.0089404.s008.docx]

**Table S7.** Pearson product-moment correlations between biomass and four phenotypic traits measured on experimental *Gilia tricolor* plants across three habitat zones spanning a local population boundary in 2010.

|  | | **Emergence Day** | | | | **Leaf Length** | | | | **Senescence Day** | | | | **Longest Internode** | | | |
| --- | --- | --- | --- | --- | --- | --- | --- | --- | --- | --- | --- | --- | --- | --- | --- | --- | --- |
|  | | ***N*** | | ***r*** | ***P*** | ***N*** | | ***r*** | ***P*** | ***N*** | | ***r*** | ***P*** | ***N*** | | ***r*** | ***P*** |
| Core | | 44 | | -0.2601 | 0.0882 | 42 | | 0.1195 | 0.4511 | 44 | | 0.0966 | 0.5328 | 44 | | 0.5914 | **<0.0001** |
| Margin | | 42 | | 0.2132 | 0.1752 | 40 | | 0.8460 | **<0.0001** | 42 | | 0.3305 | **0.0325** | 39 | | 0.5881 | **<0.0001** |
| Exterior | | 22 | | -0.0082 | 0.9712 | 17 | | 0.8493 | **<0.0001** | 22 | | 0.3650 | 0.0949 | 20 | | 0.5616 | **0.0100** |

Correlations significant at *P* < 0.05 are shown in bold.
