# Supplementary material for: Propagule Limitation, Disparate Habitat Quality, and Variation in Phenotypic Selection at a Local Species Range Boundary
Source: PLoS One. 2014 Apr 9;9(4):e89404. doi: 10.1371/journal.pone.0089404 (PMC3981700; doi:10.1371/journal.pone.0089404)
Supplement: Table S8 — Bootstrapped confidence intervals for the strength and direction of estimated total phenotypic selection ( STS ) on five traits of Gilia tricolor. (DOCX) [file pone.0089404.s009.docx]

**Table S8.** Bootstrapped confidence intervals for the strength and direction of estimated total phenotypic selection (*S_TS_*) on five traits of *Gilia tricolor.*

|  | **2008** | | | **2010** | | |
| --- | --- | --- | --- | --- | --- | --- |
|  | **Core** | **Margin** | **Exterior** | **Core** | **Margin** | **Exterior** |
| **Response** |  |  |  |  |  |  |
| Emergence Day | -0.48, 0.05 | -0.59, 0.40 | -1.02, 0.50 | 0.04, 0.11 | -0.43, 1.01 | -0.56, 0.53 |
| Senescence Day | 0.58, 1.08 | 0.09, 1.57 | -0.12, 0.55 | 0.32, 0.60 | 0.90, 1.40 | 0.29, 4.57 |
| Leaf Length | 0.58, 1.33 | 0.66, 1.74 | 0.24, 2.55 | 0.18, 0.41 | 0.58, 1.92 | 0.43, 1.94 |
| Longest Internode | -0.31, 0.57 | -0.96, 0.48 | -0.29, 1.10 | 0.44, 0.65 | 0.43, 1.38 | 0.56, 1.81 |
| Biomass | 0.77, 1.29 | 1.21, 2.02 | 0.15, 0.58 | 0.32, 0.60 | 0.90, 1.40 | 0.29, 4.57 |

Selection coefficients and confidence intervals were as calculated by separate bootstrapped separate generalized linear mixed models predicting relative fitness from standardized values of each trait, within year and habitat zone. Mean values and significance are giving in Table 2
